# Hydrogen segregation and its roles in structural stability and metallization: silane under pressure

**DOI:** 10.1038/srep13039

**Published:** 2015-08-12

**Authors:** Wenwen Cui, Jingming Shi, Hanyu Liu, Yansun Yao, Hui Wang, Toshiaki Iitaka, Yanming Ma

**Affiliations:** 1 State Key Laboratory of Superhard Materials, Jilin University, Changchun 130012, China; 2 Department of Physics and Engineering Physics, University of Saskatchewan, Saskatoon, Saskatchewan, S7N 5E2, Canada; 3 Canadian Light Source, Saskatoon, Saskatchewan, S7N 0×4, Canada; 4 Computational Astrophysics Laboratory, RIKEN, 2-1 Hirosawa, Wako, Saitama 351-0198, Japan; 5 Beijing Computational Science Research Center, Beijing 10084, China

## Abstract

We present results from first-principles calculations on silane (SiH_4_) under pressure. We find that a three dimensional *P*-3 structure becomes the most stable phase above 241 GPa. A prominent structural feature, which separates the *P*-3 structure from previously observed/predicted SiH_4_ structures, is that a fraction of hydrogen leaves the Si-H bonding environment and forms segregated H_2_ units. The H_2_ units are sparsely populated in the system and intercalated with a polymeric Si-H framework. Calculations of enthalpy of formation suggest that the *P*-3 structure is against the decomposition into Si-H binaries and/or the elemental crystals. Structural stability of the *P*-3 structure is attributed to the electron-deficient multicenter Si-H-Si interactions when neighboring silicon atoms are linked together through a common hydrogen atom. Within the multicenter bonds, electrons are delocalized and this leads to a metallic state, possibly also a superconducting state, for SiH_4_. An interesting outcome of the present study is that the enthalpy sum of SiH_4_ (*P*-3 structure) and Si (fcc structure) appears to be lower than the enthalpy of disilane (Si_2_H_6_) between 200 and 300 GPa (for all previously predicted crystalline forms of Si_2_H_6_), which calls for a revisit of the stability of Si_2_H_6_ under high pressure.

The pursuit of metallic hydrogen and its high-temperature superconductivity is of great interest to the condensed-matter community^1^,^2^. Producing metallic hydrogen in bulk, however, requires a significant static pressure that exceeds the limit of current experimental capacities. Alternative approaches may be possible, as suggested by Ashcroft^3^, that some simple hydrides, with the average valence density comparable to pure hydrogen, could become metallic at significantly reduced pressures. Similar to pure hydrogen, the metallic states of these hydrides should also have large band overlapping, high Debye temperature, and strong electron-phononcoupling (EPC) that can all lead to phonon-mediated superconductivity. According to the Goldhammer-Herzfeld criterion, Group-IV hydrides, such as methane (CH_4_) and silane (SiH_4_), are potential candidates for this behavior^3^.

At ambient temperature, SiH_4_ solidifies at around 1.7 GPa into a four-fold molecular crystal. Upon increasing the pressure, one expects higher coordinatedstructures of SiH_4_ to become stable, by virtue of multicenter bonding. Previously, several high-pressure structures of SiH_4_ have been predicted by different groups, with the metallization pressure estimated from as low as 20 GPa to above 220 GPa^4^,^5^,^6^,^7^,^8^. Some of the predicted phases have been suggested to be superconducting, as well, with the estimate of *T*_*c*_ ~ 16 K to 260 K. Experimental determinations of high-pressure structures of SiH_4_ have been carried out using various techniques. Degtyareva*et al.* observed from powder x-ray diffraction patterns that SiH_4_ remains four-fold to around 25 GPa, and likely transforms to a new phase at higher pressures^9^. Chen *et al.* observed that SiH_4_ sample turns opaque above 27 GPa, which, in combination with measured Raman and IR reflectivity, led to the suggestion that SiH_4_ metallizes near 60 GPa^10^. Eremets *et al.* later reported the metallization of SiH_4_ to occur between 50 and 65 GPa based on electrical resistance measurement, and also found this phase to be superconducting(maximum *T*_*c*_ ~ 17.5 K)^11^. The crystal structure of the metallic phase (*P*6_3_), however, was unusual: its volume shrinks nearly 60% from its precursor the *P*2_1_/*c* structure at the phase transition. According to Degtyareva *et al.*, this implies that the observed metallization actually arisesfrom contaminations in the system rather than from the SiH_4_ sample^12^. This was supported by the x-ray diffractionstudy of Hanfland *et al.*^13^ in which SiH4 was found to undergo pressure-inducedamorphization at pressures above 60 GPa recrystallizing at 90 GPainto an insulating*I*4_1_/astructure original proposed from *ab initio* calculations by Pickard and Needs^14^,^15^. At about the same time, Strobel *et al.* reported a comprehensive experimental study which confirmed Hanfland *et al.*’s observations^16^. Strobel *et al.* observed that SiH_4_ darkens above ~50 GPa, and attributed it to a partial loss of crystallinity. Above 100 GPa, SiH_4_ recrystallizes intothe *I*4_1_/*a* (or *I*-42*d*) structure, and remainsin this structure to at least 150 GPa.

In addition to pure silane, the mixtures of silane and molecular hydrogen have also been investigated. In mixtures, hydrogen molecules are perturbed by SiH_4_. Such intermolecular interactions can destabilize the covalent bonds in H_2_, showing promise of obtaining a metallic state at moderate pressures. Almost concurrently, Strobel *et al.*^17^ and Wang *et al.*^18^ reported the synthesis of novel SiH_4_–H_2_complexes under high-pressure conditions. Using power X-ray diffraction, Strobel *et al.* observedthe formation of a stoichiometric SiH_4_(H_2_)_2_ crystal near 7 GPaand identified its structure as a cubic *F*-43*m* structure. Using Raman spectroscopy, Wang *et al.* studied the behavior of SiH_4_–H_2_ fluids and established a binary eutectic point at 72 mol% H_2_ and 6.1 GPa. Above the eutectic point the fluid crystallizes in a solid solution. The SiH_4_–H_2_ phase diagrams revealed in both studies are very similar. Detailed electronic structures and chemical bonding of the SiH_4_–H_2_ mixtures have been subsequently examined through the use of first-principles methods^19^,^20^,^21^,^22^,^23^.

While recent studies revealed a great deal of information on the behavior of SiH_4_ under pressure, the jury is still out on the initial proposal. As the research progresses, the reported metallization of SiH_4_ near 55 GPa is now facing challenges. Theory suggests that SiH_4_ reaches a metallic state at a much greater pressure (220 GPa), through a phase transition to a *Pbcn* structure^8^. Motivated by the significant interests in this subject, in the present study we employ theoretical structural searches and property predictions to investigate SiH_4_ in the post-*I*4_1_/*a* region. We systematically searched for stable structures of SiH_4_ at pressures above 200 GPa, using a heuristic algorithm based on particle swarm optimization (calypso method)^24^,^25^. We predict a new structure of SiH_4_ above 241 GPa. This structure (*P*-3) is computed to be metallic and perhaps also superconducting as inferred from the electron-phonon coupling. Our prediction helps to address the phase diagram of SiH_4_ at high pressure; while its significance goes beyond. Disilane (Si_2_H_6_) is the next member in silicon hydride series (Si_n_H_2n+2_), which was suggested in theory to become stable under pressure, stable with respect to decompositions into other binaries and/or the elemental crystals^26^. Using similar argument as for SiH_4_, solid Si_2_H_6_ was predicted to be metallic and superconducting. With the addition of the *P*-3 structure in the SiH_4_ phase diagram, however, according to our convex hull calculations all previously predicted Si_2_H_6_ structures appear to be thermodynamically unstable with respect to the decomposition of Si and SiH_4_ to at least 300 GPa. This therefore suggests a revisit of previous studies on solid Si_2_H_6_ and a reconsidering of its very stability at high pressure.

## Methods

Our structure prediction is based on a global minimum search of the enthalpy surfaces obtained by *ab initio* calculations at a constant pressure, through CALYPSO (crystal structure analysis by particle swarm optimization) methodology and its same-name code^24^,^25^. For silane, disilane, silylene and lilicane, structure predictions with upto 6 formula units (f.u.) in the simulation cell were performed at 200 and 300 GPa, respectively, however, for the complex Si_5_H_18_, simulation cells containing only 1 f.u. were considered. The *ab initio* calculations were performed using density functional theory^27^ within the Perdew-Burke-Ernzerhof (PBE) parameterization^28^ of the generalized gradient approximation (GGA) as implemented in the Vienna *ab initio* simulation package (VASP) code^29^. The all-electron projector-augmented wave (PAW) method^30^ was adopted with 3*s*^2^3*p*^2^ and 1*s*^1^ as valence electrons for Si and H atoms, respectively. Planewave energy cutoffs of 500 eV and 700 eV, and uniform Monkhorst-Pack (MP) meshes^31^ for Brillouin zone (BZ) sampling with resolutions of 2π × 0.06 Å^−1^ and 2π × 0.03 Å^−1^ were employed in the structure predictions and subsequent calculations (e.g. of thermodynamic stability), respectively. The errors in the enthalpy differences of the structures studied in the structure predictions and the subsequent calculations were found to be in the order of 1 meV/atom and less than 1 meV/atom, respectively. The contribution of the electronic entropy to the enthalpy differences aroused by the MP method is less than 0.1 meV/atom, and negligible. The phonon density of states was calculated in harmonic approximation by the finite displacement method^32^ as implemented in the PHONOPY program^33^, and the vibrational free energy was estimated therefrom^34^. The underlying force constants were calculated in 2 × 2 × 1 supercells with 4 × 4 × 5 MP meshes for BZ sampling for the *P*-3 and *Pbcn* structures and a 4 × 4 × 4 MP mesh for the *I*4_1_/*a* structure. Electron-phonon coupling (EPC) calculations were carried out by using density functional perturbation theory^35^ as implemented in the QUANTUM ESPRESSO package^36^ with a kinetic energy cutoff of 80 Ry. A 16 × 16 × 12 *q*-mesh in the first BZ was used with Gaussians of width of 0.05 Ry in the EPC calculations.

## Results and Discussion

**Figure 1:**
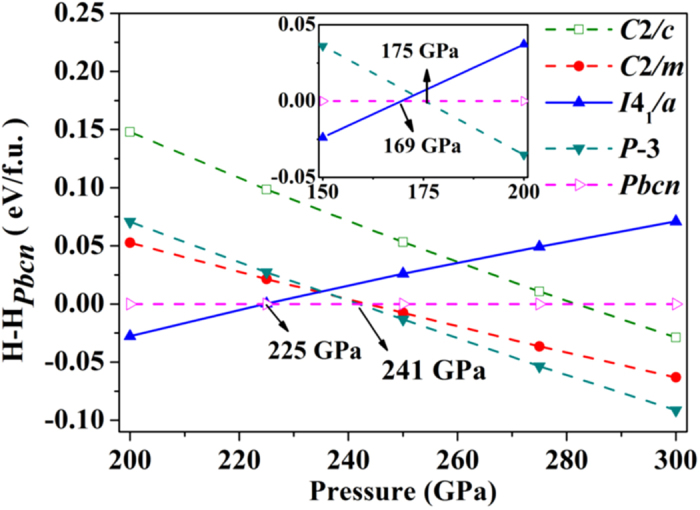
Calculated enthalpies per formula unit (f. u.) for candidate structures of SiH_4_. Enthalpies of the *Pbcn* structure (Ref. [8]) were taken as the reference enthalpies. (Inset) Relative energetics for the *I*4_1_/*a* (Ref. [14]), *Pbcn* and *P*-3 structures with the inclusion of the vibrational free energy.

In Fig. 1, the calculated enthalpies (*H* = *E* + *pV*) of the candidate structures of SiH_4_ are compared between 200 to 300 GPa. In this pressure range, our structural searches successfully predicted a new structure with the *P*-3 space group, as well as revealed the previously observed/suggested structures, *i.e.*, structures with *P*2_1_/*c*, *Fdd*2, *I*4_1_/*a*, *C*2/*c* and *Pbcn*space groups^8^,^9^,^14^. The experimentally identified *I*4_1_/*a* structure^11^,^14^,^16^ is calculated to be the most stable phase at the low-pressure end. The *I*4_1_/*a* to *Pbcn* structural transformation is calculated to occur at about 225 GPa, in a good agreement with previous study^8^. Near 241 GPa, the newly predicted *P*-3 structure becomes the most stable phase of SiH_4_. Enthalpy of the *P*-3 structure is constantly lower than that of the *C*2/*c* structure^14^, by more than 0.08 eV per formula unit. As well, we predict a metastable *C*2/*m* structure that also appears to be more stable than the *C*2/*c* structure. The optimized structural parameters of the *P*-3 and *C*2/*m* structures are listed in Table 1. To account for the temperature effects, we estimated the vibrational free energies (at 300 K) for the *I*4_1_/*a*, *Pbcn* and *P*-3 structures at four different pressures, using a harmonic method^34^ (Table 2). As shown in the inset of Fig. 1, the inclusion of the vibrational free energies does not alter the phase transition sequence, but shift down the two transition points in pressure (see explanation later). It is worth noting that the small proton mass may induce significant quantum nuclear effects, which can deviate the system from a harmonic description, and therefore, shift the two transition pressures further. A rigorous study of this should turn to explicit calculation of the free energy with anharmonic contributions from both the thermal and the quantum nuclear effects includedx^37^. This could be achieved by performing *ab initio* path-integral molecular dynamics simulations^38^, however, which is beyond the computational load we can afford nowadays. Here, we just speculate that the temperature effects will further stabilize the *P*-3 structure with respect to the other two structures. Moreover, the appearance of a molecular bonding environment in the *P*-3 structure (as will be discussed below) suggests that the van der Waals (vdW) interaction may play an important role in the phase transitions. To evaluate this effect, the enthalpies of *I*4_1_/*a*, *Pbcn* and *P*-3 structureshave been recalculated usingoptB88-vdW functional^39^,^40^,^41^,^42^. The results indicate that the vdW interaction does not change the phase transition sequence, but increase the transition pressures of *I*4_1_/*a* → *Pbcn* and *Pbcn* → *P*-3 from 225 and 241GPa to 242 and 285 GPa, respectively.

**Figure 2:**
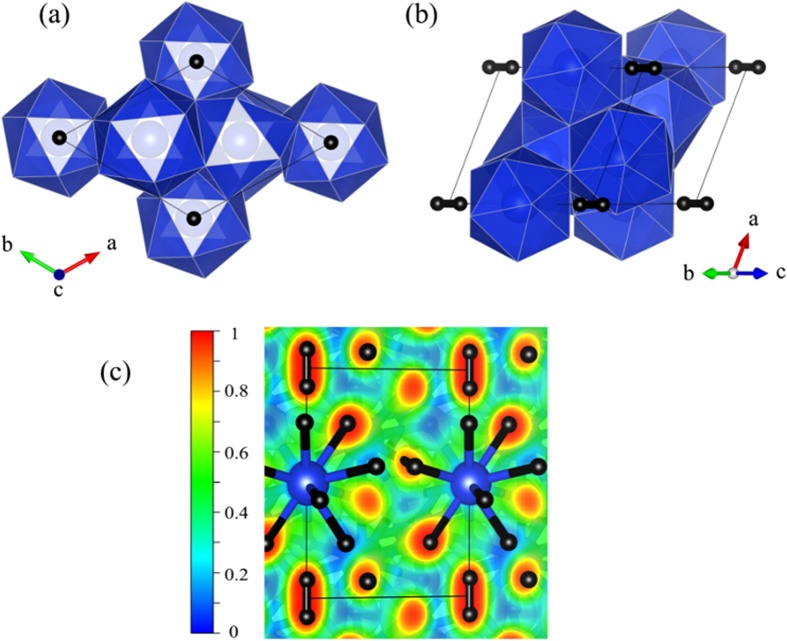
(**a**,**b**) Unit cell of the *P*-3 structure viewed from two different angles. The polymeric Si-H framework is shown as interconnected polyhedrons; Si and H atoms are located at the polyhedron centers and vertices, respectively. Isolated H_2_ units are represented by the dumbbells. (**c**) Calculated ELF map of the *P*-3 structure in the *yz* plane. Large and small balls represent the Si atoms and H atoms, respectively.

The *P*-3 structure is shown in Fig. 2(a,b) in two different views. Its unit cell contains 5 SiH_4_ formula units. The extended structure can be described as a polymeric Si-H framework intercalated by quasi-hexagonal layers of H_2_ units. Isolated H_2_ units in Group-IV hydrides are not entirely new – previous studies^43^,^44^,^45^,^46^,^47^,^48^ suggested them to exist in solid germane (GeH_4_), stannane (SnH_4_), and plumbane (PbH_4_). In SiH_4_, the H_2_ units are sparsely populated with negligible intermolecular interactions. This is in contrast to heavier Group-IV hydrides in which the H_2_ units are strongly correlated with a tendency for H_2_ – H_2_ pairing. To analyze the bonding environment, we employed the electron localization function (ELF)^49^ as a measure of relative electron localization with respect to a uniform electron gas. The ELF value represents a probability (0 to 1), with large values identifying places in the structure where electrons of opposite spins present, e.g., in cores, bonds, or lone pairs. As Fig. 2(c) shows, the intramolecular regions in the H_2_ units have a very large ELF value (close to 1) resulted from a high degree of electron paring. In fact, the ELF map of the H_2_ units is consistent to that of a gas-phase H_2_ molecule, suggesting that these H_2_ units are inact. This conclusion is resonating with the exceptionally short H-H distance in the H_2_ units, *i.e.*, 0.75 Å (calculated at 300 GPa), not so different from that of a H_2_ molecule.

In the Si-H framework, each silicon atom is coordinated to 12 nearest neighbor hydrogen atoms, which are then bridged to other silicon atoms. Overall, a third of the bridging hydrogen atoms are shared by 4 silicon atoms and the rest are shared by 3, yielding a Si_5_H_18_ stoichiometry. Such Si-H geometry goes far beyond the ubiquitous four-fold coordination of Group-IV elements, and on the face of it, one sees the enigma of silicon atoms forming more bonds (12) than the number that seems to be sufficient for their available valence electrons (4). This ‘forbidden’ increment of crystal coordination, however, is a commonplace in materials under high pressure, and can be explained by the schemes of electron-deficient multicenter bonding^50^. A quantum Atoms in Molecules (AIM) analysis^51^ (Table 3) reveals that the bridging hydrogen receive ~0.7 e^−^ per atom from neighboring silicon (which is also seen in Fig. 2(c)). In contrast, the H_2_ units almost have no charge transfer from/to the Si-H framework. At this point, silicon atoms have much reduced *s*/*p* mixing in its delocalized multicenter bonding, compared with that in molecular SiH_4_ (which favors definite coordination geometry, as we know). A similar electron-deficient scenario is also encountered in the other two candidate structures, *I*4_1_/*a* and *Pbcn*, but the difference is that in these two structures the bridging hydrogen atoms are shared by only 2 silicon atoms^8^,^14^. Each silicon atom is coordinated to 8 hydrogen atoms; pairs of which bridge to four neighboring silicon atoms, forming two three-center-two-electron (3c-2e) bonds. This bonding arrangement is a prototypical electron-deficient geometry that is commonly known for the diborane molecule (B_2_H_6_)^52^.

Two interesting observations arise from a comparison of the *P*-3 structure to the *I*4_1_/*a* and *Pbcn*structures. At first, the *P*-3 structure has higher coordination geometry which corresponds to a smaller volume. For example, at 250 GPa, the calculated volume for the *I*4_1_/*a, Pbcn*and *P-*3 structuresgradually decreases in a sequence of 13.59 Å^3^/f.u., 13.43 Å^3^/f.u. and 13.17 Å^3^/f.u., respectively. Evidently, the *P*-3 structure would have the smallest *p*V work, which provides energetic advantages. Secondly, in the *P*-3 structure the bridging hydrogen atoms are constrained by more silicon atoms. This yields reduced frequencies for the Si…H…Si bending modes in the mid-frequency range (20–70 THz, at 150 GPa), which is the main reason why the vibrational free energy of the *P*-3 structure is constantly lower than the other two structures (Table 2). In the translational region, *i.e*., below 20 THz, the vibrational free energy is mainly resulted from Si lattice modes, so it is very similar for all three structures. The *P*-3 structure also has high-frequency H-H vibron modes above 100 THz. These modes however do not have notable contributions to the vibrational free energy due to their low density of states. At 150 GPa, for example, the H-H vibrons only contribute 0.04 eV/f.u. to the vibrational free energy of the *P*-3 structure.

**Figure 3:**
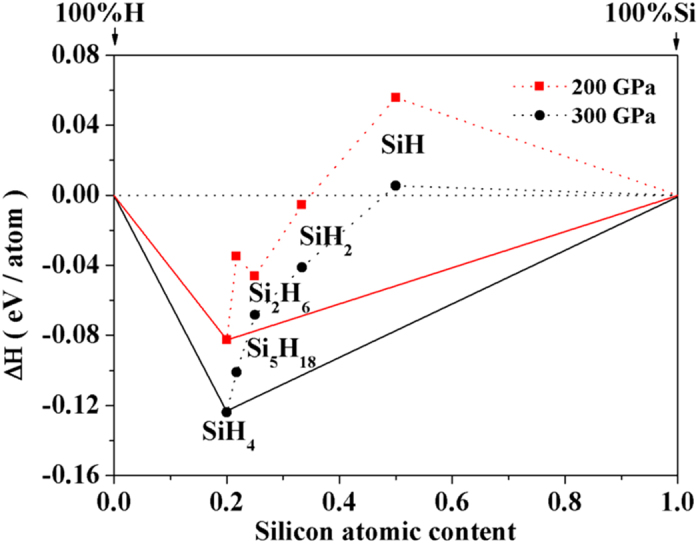
Enthalpy of formation (△*H*_*f*_, with respect to elemental crystals of H_2_ and Si) for four Si-H stoichiometries calculated at 200 and 300 GPa. The abscissa *x* represents the atomic content (%) of Si in the stoichiometry. Stoichiometry with a negative △*H*_*f*_ means that the binary phase is more stable than the elemental phases, while the convex hull of the △*H*_*f*_ values (solid lines) connects the thermodynamically stable stoichiometries.

An important issue is worthy of investigation at this point. The appearance of the H_2_ units in the *P*-3 structure, as well as the short H-H contact, seems to indicate a trend of segregation toward the Si_5_H_18_ + H_2_ limit. As crystalline SiH_4_ is known to decompose between 50 ~ 100 GPa^16^, it is not unreasonable to speculate it to decompose again at higher pressures. To this end, we examined the phase stability of the *P*-3 structure with respect to the decompositions into other possible Si-H binaries and H_2_. Four stoichiometries were used for the binaries, namely, Si_5_H_18_, Si_2_H_6_ (disilane), SiH_2_ (silylene), and SiH (silicane). To find the most stable structure of these stoichiometries, we performed additional structure searches at 200 and 300 GPa. The lowest-enthalpy structure that arises from the search was employed as the candidate for each stoichiometry. In Fig. 3, the calculated enthalpy of formation (△*H*_*f*_, with respect to elemental crystal of H_2_ and Si) is shown for all four stoichiometries. Here a negative △*H*_*f*_ means that the binary phase is more stable than the elemental crystals, while the convex hull of the △*H*_*f*_ values (solid lines) connects the stable phases; in this case they are H_2_, Si, and SiH_4_. Clearly, SiH_4_ (*P*-3 structure) is the most stable Si-H stoichiometry at 200 and 300 GPa. The △*H*_*f*_ of the *P*-3 structure relative to Si_5_H_18_ and H_2_ (*Cmca*12^53^) structure is negative which confirms its stabilities.

It is perhaps not surprising that Si_5_H_18_, SiH_2_, and SiH crystals are predicted to be less stable than SiH_4_, since these species are either reactive intermediates or not known to exist experimentally. A significant finding is that the Si_2_H_6_ crystal appears to be unstable as well, with respect to the decomposition of Si + SiH_4_ crystal. To our best knowledge, Si_2_H_6_ is the only known higher silanes that can readily be prepared in laboratory, usually by the reaction of silicon chloride (Si_2_Cl_6_) with lithium aluminum hydride (LiAlH_4_). Due to the weaker Si-Si bond (226 kJ mol^−1^), Si_2_H_6_ decomposes slowly even at room temperature^54^. On the other hand, previous theoretical studiessuggest that the stability of Si_2_H_6_ can be enhanced by applying pressure^26^,^55^. A series of crystalline Si_2_H_6_ polymorphs, with the space group *Cmcm*, *C*2/*c*, and *Pm*-3*m*, have been predicted to be thermodynamically stable and superconducting at pressures above 190 GPa. On the contrary, new calculations using the *P*-3 structure as the most stable structure of SiH_4_ show that these Si_2_H_6_ polymorphs would all have positive △*H*_*f*_with respect to the crystal of SiH_4_ + Si between 200 and 300 GPa (Table 4). This outcome therefore suggests a revisit on the stability of crystal Si_2_H_6_ at high pressures. It should be noted here that the choices of the Si-H binaries as the decomposition products are based upon the known Si-H stoichiometries observed at ambient pressure. It is possible that other Si-H binaries, in additional to what have been considered here, can be stabilized at high pressure.

It is worth noting that another recent theoretical study^56^ reports an interesting result on the disproportionation of GeH_4_ at high pressure. In this study, zero-point energies were estimated using the harmonic approximation, which is very similar to the approach adopted in the present study. Under this consideration, a new stoichiometry for the Ge-H binaries, namely GeH_3_, is predicted to become energetically stable with respect to the decomposition of elemental crystals of Ge and H_2_ near 175 GPa. The GeH_4_, on the hand, was suggested to be unstable with respect to the decomposition of GeH_3_ and H_2_. These results are distinctly different for that of the present study in which the SiH_4_ is suggested to be the most stable stoichiometry for the Si-H binaries at high pressures. Experimentally, SiH_4_ is the most stable binary in its hydride series (Si_n_H_2n+2_) at ambient pressure^54^. At high pressure, SiH_4_ decomposes near 50 GPa but recrystallizes above 100 GPa, and remains stable to at least 192 GPa, the highest pressure attempted in the experiments^11^,^16^. The *P*-3 structure is predicted to become stable near 177 GPa (with harmonic zero-point corrections), which is well within the experimental stability range for SiH_4_. The study of GeH_3_ on the other hand indicates that there are maybe new stoichiometries for this group, perhaps unprecedented at ambient pressures, may become stable at high pressures and this awaits to be discovered in future experiments.

**Figure 4:**
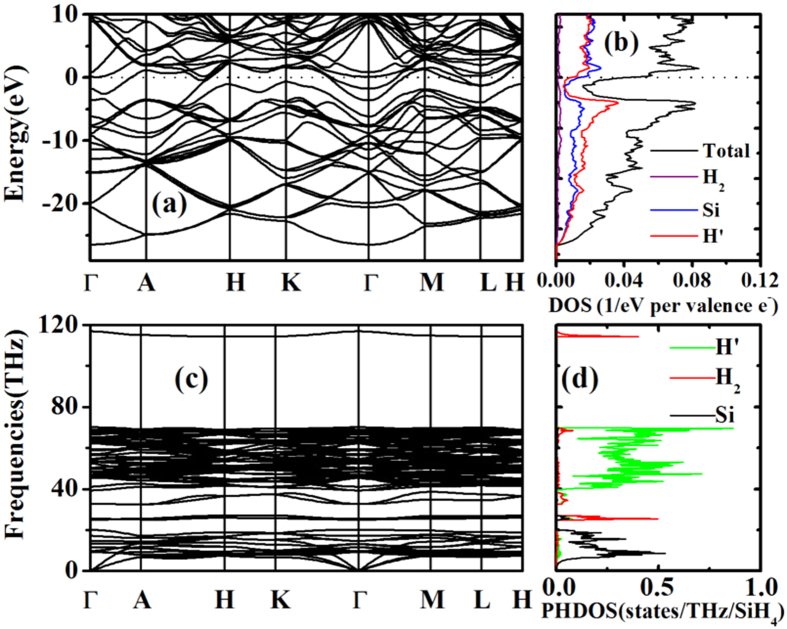
(**a**,**b**) Calculated band structure, total DOS, and site-projected DOS near the Fermi level for the *P*-3 structure at 300 GPa. The Fermi level is at the energy origin. (**c**,**d**) Calculated phonon dispersion relations and site-projected phonon DOS for the *P*-3 structure at 300 GPa. In both (**b**,**d**), H’ and H_2_ represent the bridging hydrogen and paired hydrogen, respectively.

Figure 4(a,b) show the calculated electronic band structure and projected density of states (DOS) for the *P*-3 structure at 300 GPa. It is of considerable interest that this structure is predicted to be metallic. The DOS reveals a pseudogap developed below the Fermi energy (*E*_*F*_). Similar pseudogaps were also found in calculations on high-pressure GeH_4_ and SnH_4_ but not on PbH_4_^44^,^45^,^46^,^47^,^48^. This suggests that the electronic structure of the *P*-3 structure is not free-electron-like. Since in this case the Fermi level lies in the pseudogap, the DOS at the *E*_*F*_ in the *P*-3 structure is substantially lower than the value in a free electron gas (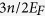, where *n* is the density of electrons), which limits the possibility of achieving high superconductivity. It perhaps merits mention that the DOS characteristics of the *P*-3 structure is similar to that of the AlH_3_ at high pressure^57^. Since Al has one less electron than Si, the Fermi level of AlH_3_ lies in the lower reach of the pseudogap which also results in a low DOS value and ultimately non-superconducting behaviors^58^. In Fig. 4b, hydrogen atoms (mostly bridging ones) contribute to the DOS throughout the entire energy range. This indicates that the hydrogen and silicon electronic states are strongly mixed in the Si-H framework. If the states near the *E*_*F*_ can be effectively coupled with the phonon modes, especially the high-frequency ones from hydrogen vibrons, a credible superconductivity can be expected^3^. However, the H_2_ units in the *P-*3 structure are populated sparsely in the lattice so their DOS and phonon DOS are both very low (Figs 4b,d), compared with their counterparts in SnH_4_, GeH_4_ and PbH_4_. We therefore do not anticipate exceptional electron-phonon coupling in the *P*-3 structure at this point.

Figure 4(c,d) show the calculated phonon dispersion relations and phonon DOS for the *P*-3 structure at 300 GPa. The absence of imaginary frequency modes suggests that this structure is mechanically stable. An outstanding feature of the ‘H_2_’ intercalation in the structure is that the vibrational modes are divided into subsets in frequency. The H_2_ pairs occupy primarily two subsets; one induced by roton modes around 25 THz, and the other induced vibron modes around 118 THz. In these two subsets, the contribution from the Si-H framework is minor, consistent with the fact that the H_2_ pairs are inactive. The Si lattice modes dominate the low-frequency subset below 20 THz, while the bridging hydrogen atoms are in the intermediate subset between 30 and 70 THz.

**Figure 5:**
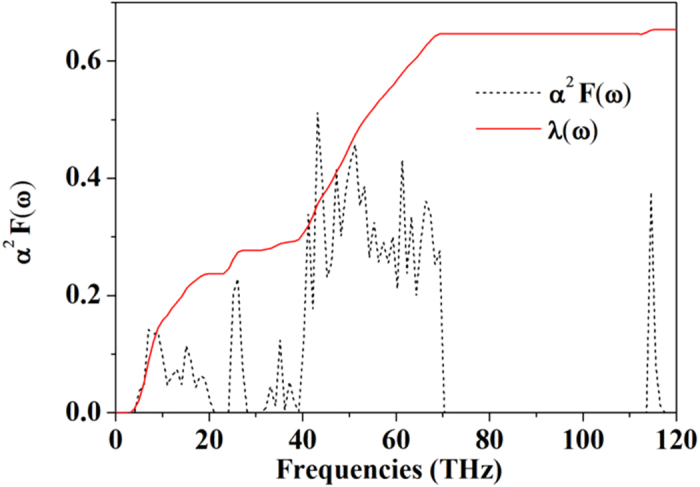
Eliashberg phonon spectral function *α*^2^*F*(ω) and integrated EPC parameter (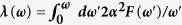) as a function of frequency for the *P*-3 structure at 300 GPa.

A brief discussion on the possibility of phonon-mediated superconductivity in the *P*-3 structure is of interest. The methodology was based on an extension of the BCS model in which the attractive, electron-phonon interaction for each phonon mode is treated explicitly. The strength of this interaction is characterized by the electron-phonon coupling parameterλ incorporating the contributions from all participating phonon modes. In Fig. 5, we present the Eliashberg phonon spectral function *α*^2^*F*(ω) and the integrated *λ* (ω) (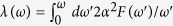) as a function of frequency ω at 300 GPa. The overall integrated *λ* is 0.63, which is a moderate value for hydrogen-rich materials and comparable to the zero-pressure value for MgB_2_^59^,^60^,^61^. The Si lattice modes contribute about 0.23 to the total *λ*. While the low frequencies of the lattice modes were considered as a disadvantage of attaining superconductivity, their reasonably strong EPC compensates it. The intermediate-frequency H modes contribute 0.36 while the high-frequency vibron modes only contribute 0.04. The logarithmic average of the phonon frequency ω_log_ is obtained as 1320 K. The electron-screened repulsive interaction is represented by the Coulomb pseudopotential μ^*^. For typical phonon-mediated superconductors, the values of μ^*^ between 0.1–0.13 are generally considered as reasonable^3^. The superconducting critical temperature *T*_*c*_ is estimated from the McMillan formula^62^ along with the Allen-Dynes correction (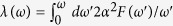)^63^, using an empirical value of μ* = 0.12. The estimated *T*_*c*_ of *P-*3 structure turns out to be 32 K at 300 GPa. A slightly improved estimate of the *T*_*c*_ of 35.1 K is made by directly solving the Eliashberg equations^2^ with the calculated *α*^2^*F*(ω), using the methodology previously implemented by us^64^. We note that these two estimates of *T*_*c*_ are both based on isotropic gap equations and an empirical value of μ*, which may not be adequate depending on the mechanism of the electron-phonon coupling in SiH_4_. A more accurate estimate of the *T*_*c*_ may be made using a fully anisotropic treatment of the gap equations, which encourages future study in this direction. Moreover, it should be pointed out that the quantum nuclear effects may deviate the system from a harmonic description and alter the estimated *T*_*c*_. As manifested by a study of AlH_3_, anharmonicity of atomic motions can cause renormalization of the vibrational modes and suppress the superconductivity^58^, On the other hand, however, anharmonic vibrations were found to enhance the electron–phonon matrix elements, as in the case of disordered materials^65^.

## Conclusions

We present a theoretical study of high-pressure phase transitions and the metallization of crystalline silane (SiH_4_). A new polymorph of SiH_4_ (space group: *P*-3) was predicted from structure searches using a heuristic algorithm based on particle swarm optimization (calypso methodology). This new phase becomes thermodynamically stable at pressures above 241 GPa, replacing the previous suggested *C*2/*c* structure in the phase diagram. The *P*-3 structure is calculated to be metallic and also superconducting with an estimated *T*_*c*_ of 32 K at 300 GPa. A unique structure feature of the *P*-3 structure is the presence of H_2_ units that are intercalated with the Si-H framework. Electron-deficient multicenter bonding along the Si…H…Si connections results in the delocalization of valence electrons and accounts for the increment of crystal coordination in the Si-H framework. Calculations of enthalpy of formation suggest that the *P*-3 structure is stable with respective to the decomposition to other Si-H binaries and/or the elemental crystals.

## Additional Information

**How to cite this article**: Cui, W. *et al.* Hydrogen segregation and its roles in structural stability and metallization: silane under pressure. *Sci. Rep.* **5**, 13039; doi: 10.1038/srep13039 (2015).

## Figures and Tables

**Table 1:** Optimized structural parameters for the *P*-3 and *C*2/*m* structures at 300 GPa.

	Lattice parameters (Å, deg)			Atomic coordinates (Fractional)		
*P*-3	*a* = 3.89	Si1	2d	0.3333	0.6667	0.3004
	*c* = 4.73	Si2	2d	0.6667	0.3333	0.1629
		Si3	1b	0	0	0.5
		H1	2c	0	0	0.0793
		H2	6g	0.3740	0.2957	0.9286
		H3	6g	0.0886	0.6624	0.5724
		H4	6g	0.7579	0.0050	0.2396
*C*2*/m*	*a* = 5.17	Si1	4g	0.5	0.2130	0
	*b* = 6.93	Si2	4i	−0.8023	0	0.8728
	*c* = 2.79	H1	8j	−0.7719	−0.1785	0.2159
	*β* = 89.5	H2	8j	−0.6255	0.1210	0.5903
		H3	4i	−0.0994	0	0.6447
		H4	4i	−0.5870	0	0.2256
		H5	8j	−0.6007	−0.3273	0.4034

**Table 2:** Calculated vibrational free energies (eV/f. u.) of the *I*4_1_/*a*, *Pbcn*, and *P*-3 structures.

Pressure(GPa)	*I*4_1_/*a*	*Pbcn*	*P*-3	△1	△2
150	1.322	1.251	1.126	−0.196	−0.125
200	1.404	1.339	1.233	−0.171	−0.106
250	1.472	1.408	1.326	−0.146	−0.082
300	1.533	1.472	1.406	−0.127	−0.066

△1 (△2) is the energy difference between the *P*-3 structure and the *I*4_1_/*a* (*Pbcn*) structure.

**Table 3:** Bader charges of the *P*-3 structure calculated at 300 GPa.

Atom	Bader Charge (e^−^)
Si1	−2.7715
Si2	−2.6169
Si3	−2.5131
H1	+0.0307
H2	+0.7225
H3	+0.7606
H4	+0.7198

Negative (positive) sign indicates an electron loss (gain) for the particular atom.

**Table 4:** Enthalpies of formation △*H*_*f*1_ of Si_2_H_6_ with respect to crystals of SiH_4_ and Si (3/2 SiH_4_ + 1/2 Si) and △*H*_*f*2_ with respect to SiH_4_, Si and H_2_ (SiH_4_ + Si + H_2_) at 200, 250 and 300 GPa.

Pressure (GPa)	Si_2_H_6_	SiH_4_	Si	H_2_	Δ**H_*f*1_ (eV/Si_2_H_6_)	Δ*H*_*f*2_ (eV/Si_2_H_6_)
200	*Cmcm*	*I*4_1_/*a*	*Fm*-3*m*	*C*2*/c*	0.249	0.042
250	*Cmcm*	*P*-3	*Fm*-3*m*	*C*2*/c*	0.256	0.016
300	*C*2*/c*	*P*-3	*Fm*-3*m*	*Cmcm*12	0.383	0.072
300	*Pm-*3*m*	*P*-3	*Fm*-3*m*	*Cmcm*12	0.388	0.077

The *C*2/*c* and *Pm-*3*m* structures of Si_2_H_6_ were previously predicted in Ref. 55, while the *Cmcm* structure predicted in Ref. 26.
